# *Correia*, *Diamond* and the *Chester* Exception: Vindicating Patient Autonomy?

**DOI:** 10.1093/medlaw/fwab016

**Published:** 2021-06-23

**Authors:** Louise Austin

**Affiliations:** School of Law, Cardiff University, Cardiff, UK

**Keywords:** Autonomy, Causation, Chester, Consent, Decision-making, Disclosure

## Abstract

In *Chester v Afshar* [2004], the House of Lords stated they were departing from the traditional rules of causation in order to vindicate the patient’s right of autonomy. Subsequent judgments in the Court of Appeal expressed concerns over the lack of clarity of the legal principles to be derived from that judgment. In *Correia* v *University Hospital of North Staffordshire NHS Trust* [2017] and *Diamond v Royal Devon and Exeter NHS Foundation Trust* [2019], however, the Court of Appeal sought to clarify the scope and limits of *Chester*. This commentary sets out the scope and limits of *Chester* in light of those judgments and considers the extent to which they can be said to be vindicating patient autonomy. Drawing upon Coggon’s typology of autonomy, it concludes that future judgments should utilise that typology to explicate which understanding of autonomy they are seeking to protect.

## INTRODUCTION^1^

Medical law roots the requirement of informed consent in the need to respect patient autonomy,^2^ but claims relating to inadequate informed consent are situated in the tort of negligence.^3^ This means that the question for the court is not the extent to which the patient’s autonomy has been respected, but whether the healthcare professional has breached their duty of care in failing to disclose information and if, as a consequence of that failure, the patient has suffered harm. The focus of such claims is usually on harm in the form of the materialisation of a risk of surgery and so the claimant has to establish a link between the non-disclosure and the materialisation of the risk.^4^ Prior to *Chester v Afshar*,^5^ this link was based on an assertion that ‘but for’^6^ the negligent non-disclosure, the patient would not have participated in the treatment in question.^7^ Ms Chester’s case differed, however, because she could not say she would not have had the surgery at all but instead said that she would not have had it when she did as she would have sought opinions from other surgeons.^8^ In giving judgment in the *Chester* case, the majority of the House of Lords held that in order to vindicate her right of autonomy, they would depart from ‘conventional causation principles’^9^ and awarded Ms Chester damages for the non-disclosure and associated materialisation of the risk. Therefore, whilst the focus of the court in informed consent cases is on the elements of negligence, rather than autonomy, *Chester* demonstrates the courts’ willingness to flex the requirements of negligence when they think it is necessary to protect patient autonomy.

The decision in *Chester* provoked debate in the literature with some commentators welcoming the decision and others critiquing the House of Lords approach.^10^ As discussed in ‘The *Chester* Exception’ section of this commentary, *Chester* was also met with criticism in the Court of Appeal which suggested that the scope and limits of the *Chester* exception were unclear.^11^ However, as discussed later in this commentary, the Court of Appeal’s judgments in *Correia v University Hospital of North Staffordshire NHS Trust*^12^ and *Diamond v Royal Devon and Exeter NHS Foundation Trust*,^13^ have shed light on the nature of the *Chester* exception. These cases illustrate that the Court of Appeal has interpreted the exception as a narrow one, limited to those cases where the claimant can show that, if adequately informed, they would have delayed the treatment in question. Given that the purpose of the exception was to vindicate patient autonomy, however, this commentary also explores whether the *Chester* exception (as interpreted by the Court of Appeal) achieves that. Drawing upon Coggon’s typology of autonomy as ideal desire, best desire, and current desire autonomy,^14^ I conclude that the *Chester* exception respects ideal desire autonomy which reflects actions according with an objective set of values. This, however, is inconsistent with the principle that patients can refuse treatment for irrational reasons, or for no reason at all. As Purshouse states, the right to refuse treatment for irrational reasons ‘cannot possibly reflect ideal desire [which] requires decisions to be rational’.^15^ Yet, the alternatives of current or best desire autonomy also have difficulties associated with their use in this context. I suggest that in future cases, courts considering questions of informed consent should utilise Coggon’s typology of autonomy in order to explicate which understanding of autonomy is engaged, enabling the problems identified with the different concepts to be acknowledged and addressed.

## THE *CHESTER* EXCEPTION

*Chester v Afshar* concerned a claim brought in negligence by Ms Chester against her surgeon, Mr Afshar. Mr Afshar recommended she undergo spinal fusion surgery to address severe back pain. The surgery was performed 3 days later. However, prior to the surgery, Mr Afshar had failed to warn her of a 1–2% risk of nerve damage resulting in paralysis. Post-operatively, the risk materialised and Ms Chester brought a claim in negligence against Mr Afshar based upon his failure to disclose the risk.^16^ Ms Chester did not say that had she been warned of the risk she would not have undergone the surgery at all but did say that as she had access to private healthcare insurance, she would have sought a second (and possibly) a third opinion. She would, therefore, have not had the surgery when she did. Had the surgery been performed on another day then, statistically, it was unlikely the risk would have materialised as there was nothing specific to her condition that made the risk more likely to occur. However, the likelihood of the risk occurring would have been the same whenever and whoever performed the surgery and so, whether she had the surgery on that day or another, she would have been exposed to the same *degree* of risk.

At first instance, Ms Chester’s claim in relation to informed consent succeeded and that decision was upheld by the Court of Appeal. Mr Afshar, therefore, appealed to the House of Lords who said the question on appeal was whether ‘the conventional approach to causation in negligence actions should be varied where the claim is based on a doctor’s negligent failure to warn a patient of a small but unavoidable risk of surgery when […] such risk eventuates but it is not shown that, if duly warned, the patient would not have undergone surgery [at all]’.^17^ As noted in the introduction to this commentary, prior to *Chester*, cases alleging inadequate informed consent had asserted that ‘but for’ the negligent failure of disclosure, the patient would not have undergone the treatment at all.^18^ The House of Lords found in favour of Ms Chester by a majority of three-two concluding that ‘justice require[d]’^19^ that her claim be allowed to succeed because ‘her right of autonomy and dignity can and ought to be vindicated by a narrow and modest departure from traditional causation principles’.^20^ The decision, however, was not without its controversy because of this stated departure from conventional approaches to causation.

Stapleton argues that no such departure was necessary because (1) Ms Chester’s claim met the ‘but for’ test as, on the balance of probabilities, the risk would not have materialised on another day, and (2) her injury was within the scope of Mr Afshar’s duty to warn as it was the materialisation of the risk about which she should have been warned.^21^ Green, however, rejects this saying that the ‘but for’ test was construed too narrowly in *Chester* and the question is not whether the injury would have occurred on the day it did, but whether it ‘would have occurred *at all* but for the defendant’s failure to warn’.^22^ Ms Chester could not say that she would not have exposed herself to the risk *at all* and so causation was not made out. However, Green goes on to argue that departure from the traditional principles of causation was not justified. In relation to their Lordships stated justification of the need to protect the patient’s right of autonomy, Green argues this is achieved through the imposition of the duty to warn. If the court finds that a breach of that duty did not violate that right, it does not mean the duty is hollow but simply acknowledges the fact that, on this occasion, the breach did not cause the patient harm.^23^

Green’s concerns about the judgment in *Chester* were echoed by the Court of Appeal in *Duce v Worcestershire Acute Hospitals NHS Trust*.^24^ Leggatt LJ described the decision in *Chester* as ‘problematic’^25^ because: (i) the failure to warn had not exposed Ms Chester to a risk she was unwilling to accept;^26^ (ii) the purpose of the duty to warn was to enable the patient to decide whether the risk was unacceptable and it was not Ms Chester’s position that the risk was one she would not have accepted at all;^27^ and (iii) the departure from traditional causation principles was justified by reference to the need to vindicate the patient’s right to make an informed choice but this ‘is not a right that is traditionally protected by the tort of negligence’.^28^ Leggatt LJ, therefore, suggested that *Chester* ‘may be thought ripe for further consideration by the Supreme Court when the opportunity arises’.^29^ Although it is worth noting that the Supreme Court had the opportunity to consider *Chester* in *Montgomery v Lanarkshire Health Board* but declined to do so, instead overturning the trial judge’s findings on causation.^30^

Whilst the Court of Appeal have confirmed that *Chester* does not create loss of autonomy as a freestanding head of damage,^31^ and that it is not authority for the proposition that a doctor will be liable for an injury caused by the materialisation of a negligently non-disclosed risk ‘without more’,^32^ judges have struggled to articulate what the something ‘more’ is. In *Meiklejohn v St George’s Healthcare NHS Trust*, Rafferty LJ said that she could not ‘identify within [*Chester*] any decision of principle’.^33^ In *Correia* and *Diamond*, however, the Court of Appeal took a different approach, seeking to clarify the scope of the *Chester* ‘exception’ and what a claimant would need to prove in order to rely on it.

## *CORREIA V UNIVERSITY HOSPITAL OF NORTH STAFFORDSHIRE NHS TRUST* [2017] EWCA Civ 356

### A. Background

Ms Correia suffered with a recurrent neuroma (a benign tumour in the nerve tissue) in her right foot and underwent surgery to remove this. The operation involved three stages with the purpose of the third stage being relocation of the nerve with a view to preventing the neuroma recurring. Unfortunately, the surgeon performing the operation, Mr Rayatt, negligently omitted the third stage and post-operatively, Ms Correia developed chronic regional pain syndrome in her right foot. She brought a claim against the Trust where the surgery had been performed alleging (i) negligent performance of the procedure, and (ii) negligent informed consent. Her case was heard at first instance in Manchester County Court and both claims failed.^34^ The Recorder held that whilst the operation had been negligently performed due to the omission of the third stage and the neuroma had probably reformed, Ms Correia had not proven that was the cause of her ongoing pain. Ms Correia appealed this decision to the Court of Appeal.

### B. Issues on appeal

In relation to the informed consent claim, Ms Correia appealed on the grounds that her consent had been negligently obtained because:

1. She had consented to a three-stage procedure and this was not what was performed;
2. She was not informed of the material risk of the neuroma reforming if the third stage of the procedure was omitted; and
3. On the basis of *Chester* if there was a failure to disclose information about the procedure, then she did not have to show that failure *caused* the damage, as long as the injury was within the scope of the duty to warn.^35^

### C. Court of Appeal’s Judgment

Ms Correia’s appeal was unsuccessful and the allegations relating to informed consent were rejected. Although a two-stage procedure had been performed instead of the intended three-stage approach, Simon LJ (who gave judgment on behalf of the court) noted that the omission of the third stage had not altered the nature of the procedure and so it did not vitiate consent.^36^ This seems to be a straightforward application of *Chatterton v Gerson*.^37^ The failure to discuss the consequences of not performing the third stage did not amount to negligent non-disclosure because the surgeon had intended to perform the third stage.^38^ Although the *Chester* point did not have to be addressed in light of the finding that disclosure had been adequate, Simon LJ did set out his interpretation of *Chester’s* ratio. He noted that the *Chester* exception was intended to be narrow and applied:

If there has been a negligent failure to warn of a particular risk from an operation and the injury is intimately connected to the duty to warn, then the injury is to be regarded as being caused by the breach of the duty to warn and this is to be regarded as a modest departure from established principle of causation.^39^

However, Simon LJ went on to say that in order to ‘rely on the exceptional principle of causation established by *Chester v Afshar*, it is necessary to plead the point and support it by evidence’.^40^ As Ms Correia had not asserted that she would have either refused the operation, or deferred it, if informed of the consequences of omitting the third stage, she could not rely on the *Chester* exception.

## IV *DIAMOND V ROYAL DEVON AND EXETER NHS FOUNDATION TRUST* [2019] EWCA Civ 585

### A. Background

Ms Diamond had an extensive history of chronic back pain and underwent spinal fusion surgery. Post-operatively, she developed an abdominal hernia which was subsequently surgically repaired by Mr Wajed by way of insertion of a mesh with abdominal wall repair. Following the hernia repair, her symptoms of abdominal pain and swelling continued and she underwent further surgery during which the mesh was removed and the hernia repaired with a single stitch. Prior to this, she was informed that due to the presence of the mesh, it was unlikely that she would be able to carry a child and so it would be inadvisable to become pregnant. As a consequence of the surgeries and their after-effects, Ms Diamond suffered with depression and anxiety.

Ms Diamond brought a claim against the Trust where the surgeries were performed alleging a negligent delay in diagnosing and treating the hernia and negligent informed consent in respect of the mesh repair. At trial, the court found there had been a negligent 2-month delay in identifying the hernia, for which Ms Diamond was awarded damages. The claim in respect of informed consent, however, failed.

Ms Diamond had alleged negligence in respect of her informed consent to the mesh repair on the basis that Mr Wajed had:

1. Failed to discuss athe alternative of a primary suture repair; and
2. Failed to discuss the implications for pregnancy of a mesh repair.

The trial judge, HHJ Freedman, found that Mr Wajed had breached his duty of disclosure in failing to discuss both the alternative of a primary suture repair and the implications of a mesh repair for pregnancy. Had this information been given, Ms Diamond should have been informed that:

1. There was a 50% chance of a primary suture repair failing within 2 years and it would be likely to fail at some point in any event.
2. Pregnancy would strain a primary suture repair increasing its chance of failure.
3. Mr Wajed strongly recommended a mesh repair and 95% of surgeons would make the same recommendation.
4. Future pregnancies would not be completely contraindicated with a mesh repair but there would be some risks:
  - The mesh *may* restrict the growth of the uterus leading to early delivery;
  - If an emergency caesarean-section was required, access to her abdomen may be difficult in the presence of the mesh;
  - After pregnancy the mesh and abdominal wall may be disrupted.
  - It would be prudent, therefore, to consult a gynaecologist if pregnancy was contemplated.^41^

Ms Diamond’s evidence was that had she been given this information, she would have elected to have a primary suture repair as she needed to have something done to relieve her symptoms but would not have wanted to compromise her ability to have children. Ms Diamond said that she regarded having children as an important part of her womanhood and had seen the impact of a hysterectomy on her sister and cousin.^42^ The risk of recurrence of the hernia would not have deterred her from undergoing a suture repair as she had accepted serious risks associated with the spinal fusion procedure.^43^

The trial judge found that Ms Diamond was a credible and truthful witness but that subsequent events had influenced her view as to what she would have done. Whilst she honestly believed she would have opted for a suture repair, he found she would have proceeded with a mesh repair for the following reasons:

1. The suture repair was likely to fail;
2. The mesh repair had a high chance of success;
3. Mr Wajed’s advice would have been that she should undergo a mesh repair and he would have been reluctant to perform a suture repair;
4. She would not have been told she *could not* have children, but that pregnancy would have additional risks;
5. Pregnancy was not in her immediate contemplation at that time as she was single, although she had thought about having a child with a previous partner 2 years earlier;
6. In these circumstances, it would have been irrational to opt for a suture repair and she was not a person who would act irrationally.^44^

Ms Diamond’s counsel then made two further arguments:

1. That she should be entitled to compensation for the ‘shock’ in discovering she could not have children.
2. That a negligent non-disclosure of information can in itself create a right to damages based on *Montgomery* and *Chester*.^45^

The first additional argument was dismissed on the grounds that it had no basis in fact or law. The duty was to inform of the risk of complications, not to advise that if she had the mesh repair she may not be able to have children in the future and so any ‘shock’ due to Ms Diamond learning that it was inadvisable to have children was too remote from the breach. In addition, she was already suffering from anxiety and depression at the time she was given the advice about pregnancy and it would be too difficult to measure the extent of any additional injury. Finally, ‘shock’ alone could not give rise to damages.^46^

In relation to the second additional argument, neither *Montgomery* nor *Chester* were authority for the proposition that a failure to warn without more gave rise to a free-standing claim for damages. *Chester* does not relieve the claimant of the obligation to prove conventional causation as a result of a failure to provide informed consent. Citing *Correia* the judge went on to find that Ms Diamond could not come within the *Chester* exception because even if being told she could not carry a child was an injury, it was not ‘intimately connected to the duty to warn’.^47^ Ms Diamond appealed to the Court of Appeal.

### B. Issues on Appeal

Ms Diamond appealed on three grounds:

1. The judge incorrectly applied a test of rationality when determining whether she would have accepted or rejected the mesh repair if properly informed.
2. The judge was wrong to reject her psychiatric injury claim. She was shocked to learn of the pregnancy related risks not previously disclosed, and it was foreseeable that in such circumstances, she may suffer shock, distress, and consequential depression.
3. If she could not recover for psychiatric injury on conventional principles, then she was entitled to succeed on the basis of the *Chester* principle as described in *Correia* that the injury was ‘intimately connected’ to the duty to warn.^48^

### C. Court of Appeal’s Judgment

Ms Diamond’s appeal was unsuccessful with Davies LJ giving judgment for the court.

#### 1. Ground 1: incorrectly applying a test of rationality

On behalf of Ms Diamond it was argued that as the judge had found her to be a credible and truthful witness, he should not have rejected her assertion that she would have refused the mesh procedure in favour of the suture repair on the grounds that to have done so would have been irrational.

Davies LJ said the judge had asked the right question: what would Ms Diamond have done armed with the knowledge of the pregnancy risks associated with a mesh repair and the advice that a suture repair would almost probably fail? His assessment of this question took account of the relevant clinical facts as well as the claimant’s circumstances and the potential influence of hindsight. HHJ Freedman took account of the reasonable person in the patient’s position, whilst giving weight to Ms Diamond’s characteristics.^49^ On the evidence, the judge was entitled to conclude that opting for a suture repair would be irrational and that she was not someone who would behave irrationally.^50^

#### 2. Ground 2: rejection of psychiatric injury claim

The advice that it was unlikely that Ms Diamond would be able to carry a child was given to her by another surgeon, Mr Jones. It was Mr Jones’ advice, rather than anything said or done by Mr Wajed, that triggered or exacerbated her psychiatric injury. It was not foreseeable that the failure to disclose pregnancy risks prior to the mesh surgery would lead to Ms Diamond being told by another doctor 3 years later that she could not carry a child. The claim for psychiatric injury had been rightly dismissed.^51^

#### 3. Ground 3: an intimate connection between the injury and the duty to warn

The judge found she would have proceeded with the mesh repair in any event. There was no medical evidence to support the contention that Mr Wajed’s advice was the cause of any trigger or exacerbation of psychiatric injury. In light of this, it could not be said that the non-disclosure was ‘intimately connected’ with the later alleged psychiatric injury.^52^

Whilst the judgments in *Correia* and *Diamond* address more than the *Chester* exception, the following section focuses upon what those decisions suggest about the scope and limits of *Chester*.

## SCOPE AND LIMITS OF CHESTER

*Correia* and *Diamond* suggest that *Chester* is confined to cases where, had the claimant been given the required information, they would not have undergone the treatment when they did, albeit that they may have undergone the treatment at a later date. Further, the ‘intimate connection’ between the failure to disclose and the injury which is the subject of the claim appears to be the materialisation of a risk of harm, exposure to which could have been deferred with proper disclosure. Therefore, there are three possible scenarios that may arise in respect of non-disclosure of information and its effect upon a patient:

1. The information would not have altered the patient’s decision and treatment would have proceeded as it did;
2. The information would have altered the patient’s decision and they would have *refused* the treatment in question; or
3. The information would have altered the patient’s decision and they would have *delayed* treatment.^53^

In the first scenario, causation will not be made out and the *Chester* exception will not apply. In the second scenario, causation will be made out in accordance with conventional principles. In the third scenario, the Chester exception applies. However, in either scenario two or three, the claimant must clearly plead their case, setting out the reasons why they would have refused or delayed the treatment. This reflects a subjective approach to causation. *Diamond* then confirms that the credibility of the claimant’s subjective evidence will be assessed by reference to what a *reasonable* patient would have done.^54^ If the intention of the House of Lords (now the Supreme Court) was to utilise legal principle to safeguard the patient’s right of autonomy, does this interpretation of *Chester* achieve this?

## VINDICATING PATIENT AUTONOMY?

The extent to which the Court of Appeal’s interpretation of the *Chester* exception does, or does not, respect patient autonomy, depends upon which concept of autonomy is employed. The literal translation of ‘autonomy’ from its Greek origins means ‘self-rule’,^55^ suggesting that what matters is the extent to which someone can be said to have reached their own decision. However, there is a vast body of ethical literature which builds on this simple starting point and engages with the notion of what it means to be acting autonomously. There is, however, no shared understanding and Coggon’s typology of autonomy identifies three broad understandings of autonomy present in the ethical literature.^56^ I draw upon this framework in order to assess the extent to which the Court of Appeal’s interpretation of the *Chester* exception protects patient autonomy. The three understandings are ideal, best, and current desire autonomy:

1. *Ideal desire autonomy* refers to actions which reflect what a person *should* want, as measured by reference to a universal or objective standard of values.
2. *Best desire autonomy* refers to actions which reflect a person’s overall desire in light of their values, even if it does not reflect their immediate desire.
3. *Current desire autonomy r*efers to an action which reflects a person’s immediate inclination without further reflection.^57^

Ideal and best desire autonomy both envisage the need for reflection before a person commits themselves to an action, in order to determine whether the action reflects what that person *should* want or their *overall* desires. Current desire autonomy, however, does not require such reflection.^58^

We have seen that the positioning of informed consent in the tort of negligence means that the claimant will only have their right of autonomy vindicated through a finding of liability if they can show that the non-disclosure caused them to reach a different decision (refusal of, or delaying, treatment) to the one they would have made had adequate disclosure taken place. This involves the claimant explaining *why* that information would have altered their decision. Thus, the court does not accept the claimant’s immediate inclination as to what they would have done (current desire autonomy) but requires them to reflect upon that information and justify their decision in light of their value commitments (best desire autonomy). Even then, the court may still reject the claimant’s evidence if, upon assessment of its credibility by reference to the reasonable patient (ideal desire autonomy), the court concludes that the claimant would not have made a different decision. This is well illustrated in the case of *Diamond*.

Ms Diamond did not limit her evidence to saying she would have rejected the mesh repair in favour of the suture repair (current desire autonomy). She went further, drawing upon her attitude towards her ability to conceive and carry a child to justify her decision (best desire autonomy). The court, despite finding her a credible witness, concluded she would *not* have altered her decision because, applying an objective standard of rationality (ideal desire autonomy), it would have been irrational to do so in light of the advice that a primary suture repair would almost certainly fail. The court qualifies its use of an objective standard by reference to the patient’s subjective views, finding that she was not a person who would have acted irrationally (best desire autonomy). The court, therefore, appears to be utilising a combined application of best and ideal desire autonomy. However, whilst the primary suture repair carried a high risk of failure, Ms Diamond’s assertion was that her strong desire to maintain her ability to carry a child would have outweighed the risk of failure in her assessment of how she wished to proceed. From this perspective, her decision to proceed with a suture repair is not irrational, simply a different weighting of values. Therefore, by focusing upon what a rational person would have done and then concluding Ms Diamond would not have wanted to behave irrationally, the court prioritises ideal desire autonomy *masked* as best desire autonomy.

This echoes the approach taken by the trial judge, Robert Taylor J, in *Chester*. He concluded that given Ms Chester’s aversion to surgery and concerns about being paralysed, she would not have agreed to surgery taking place in 3 days’ time if warned of the risk of paralysis (best desire autonomy). Instead, the judge accepted her assertion that she would have sought opinions from other doctors and so the operation would not have proceeded when it did. The judge then noted that he was alert to the risk of giving weight to assertions made by a claimant *after* the outcome of the surgery is known, but that applying the test of a reasonable person in her position (that is, someone with an aversion to surgery), the outcome was the same (ideal desire autonomy).^59^ Unlike *Diamond*, in *Chester* the judge took the view that the reasonable person would have acted in the same way as Ms Chester did. However, if he had taken the opposite view, Ms Chester would not have been successful in her claim on the basis of an application of ideal desire autonomy.

Coggon argues that the use of multiple understandings of autonomy allow courts to reach different outcomes on similar facts, whilst claiming that each outcome respects autonomy.^60^ The approach of the courts in *Diamond* and *Chester* illustrates that multiple understandings of autonomy can appear within the same judgment, making it difficult to determine which understanding tips the scales. At first sight it is best desire autonomy—Ms Diamond’s desire to behave rationally and Ms Chester’s aversion to surgery and concern about a particular risk. However, on closer examination it is ideal desire autonomy which carries the day. Coggon is not critical of the use of different understandings of autonomy within medical law as long as the court is explicit about which concept of autonomy is being used, although he expresses a preference for best desire autonomy.^61^ Purshouse, in contrast, prefers current desire autonomy. He argues against using ideal and best desire autonomy in medical negligence claims on the grounds that both focus on the notion of what a patient *should* want and are, therefore, paternalism masked as autonomy and undermine the principle that patients should be allowed to refuse treatment for irrational reasons, or for no reason at all.^62^

Whilst at first sight, the use of current desire autonomy is attractive, further reflection suggests some difficulties. Current desire autonomy is centred on the patient’s immediate inclination as to what action to take. In the case of non-disclosure of information, the court is trying to assess what the patient *would* have done if adequately informed but the patient is having to answer that question after the treatment in question has taken place. On the application of current desire autonomy, the claimant is being asked to express their immediate inclination about treatment in light of all the information they have. Thus, it is inevitable that this will include the claimant’s knowledge of the materialisation of the risk. This is contrary to the court’s approach which is to focus on what the claimant should have known *before* the treatment and materialisation of the risk, without the benefit of hindsight. This links to one of the compensatory aims of tort which is to put claimants in the position they would have been in but for the negligence. To illustrate that the claimant’s view is not coloured by hindsight, the claimant has to set out the reasons why they would have refused or deferred the procedure. This allows the court to assess the credibility of that evidence. It is, however, inconsistent with the application of current desire autonomy which does not demand reflection upon the reasons for a decision, or require the exclusion of particular information from decision-making. The requirement for the claimant to reflect upon and justify how and why their past self would have reacted to the information that should have been disclosed seems more aligned with best desire autonomy, and the closer the claimant’s assessment is to their overall values, the more likely the court is to find the claimant’s evidence is credible.

Best desire autonomy, therefore, may seem an acceptable compromise allowing the court to test the credibility of the claimant’s evidence (as current desire autonomy does not), without demanding the claimant’s decision is objectively rational (as ideal desire autonomy does). However, this would mean that different understandings of autonomy would be in play within the area of consent to treatment. Given that the law states that patients can refuse treatment for irrational reasons, or for no reason at all (reflecting current desire autonomy), utilising best desire autonomy in relation to causation involves the use of a more demanding concept of autonomy then is otherwise required. Purshouse suggests that using different concepts of autonomy is unacceptable and the law should be consistent.^63^ In contrast, Coggon believes employing different concepts in different contexts is acceptable, providing judges are explicit as to which concept of autonomy is engaged.^64^

I have outlined some of the difficulties with utilising current desire autonomy in respect of causation following a negligent non-disclosure of information, and some of the benefits best desire autonomy offers. However, even with those benefits, best desire autonomy may not offer a complete answer as it does not tell us how to manage conflicting values such as those present in *Diamond* where her desire to be pain free, conflicted with her desire to maintain her ability to carry children. The *Chester* exception itself arises from a conflict between Ms Chester’s long-term desire to resolve her pain, versus her aversion to surgery and risk. The presence of both meant she could not say she would never have had the surgery at all. Reverting to ideal desire autonomy does not resolve these conflicts as it may lead to a conflicting value being disregarded without explanation. This was the case in *Diamond* when the court concluded a rational person would have proceeded with the mesh repair by reference to the chances of success versus failure, but without any reference to the desire to conceive and carry a child.

These difficulties are exacerbated by the court’s failure to state what notion of autonomy is being protected. Had the court in *Diamond* explicated which understanding of autonomy was engaged, it may have ensured that conflicting values (such as the desire to conceive and carry a child) were not overlooked. Coggon’s typology offers a framework to enable judges to identify which concept of autonomy is intended to be respected without having to engage in extensive discussion of the ethical literature, given that they are judges of law not ethics.^65^

## CONCLUSION

*Chester* was framed by the House of Lords as a departure from conventional causation principles in order to vindicate the patient’s right of autonomy. In subsequent Court of Appeal cases, the *Chester* exception has had little success with Leggatt LJ commenting that the right of autonomy is not a right traditionally protected through the tort of negligence and Rafferty LJ stating that she could not identify any decision of principle within *Chester*. However, in *Correia* and *Diamond*, whilst the Chester exception was still not successful, the judgments of the Court of Appeal in those cases do offer insights into the scope and limits of the exception. Thus, it seems that the *Chester* exception applies where the claimant may not have refused treatment altogether but would have, at least, delayed it. To rely on the exception, however, the claimant must do so explicitly, setting out the reasons why they would have delayed treatment. The credibility of that evidence will then be assessed by reference to what a reasonable patient would have done.

The Court of Appeal’s approach appears to achieve the House of Lords aim of protecting the patient’s autonomy through a combination of best and ideal desire autonomy. However, on closer examination it is apparent that it is really an application of ideal desire autonomy because a patient can only be compensated for non-disclosure of information if they can offer a convincing account of their reasons as tested against an objective standard. Whilst Purshouse has argued that current desire autonomy should be the standard of autonomy in medical negligence cases, in questions of causation in non-disclosure of information claims, current desire autonomy presents difficulties as the court’s assessment of credibility demands reflection and reasons. Best desire autonomy, therefore, may seem to be an acceptable compromise but its use does not tell us how to manage conflicting values influencing decision-making.

If judges are to justify the imposition of legal standards by reference to concepts such as autonomy then, as Coggon says, it is important that judges also engage with an explication of what understanding of autonomy is intended to be respected. If judges were to do this utilising Coggon’s typology, this wouldenable judges to identify the potential difficulties highlighted here and consider possible solutions.

## Footnotes

1 This commentary draws upon cases and concepts discussed In: Louise Austin (2020) *Towards a Coherent Model of informed Consent*, University of Bristol.

2 *Chester v Afshar* [2004] UKHL 41 [18, 77]; *Montgomery v Lanarkshire Health Board* [2015} UKSC 11 [108].

3 *Chatterton v Gerson* [1981] QB 432, 443.

4 For example, in the key appellate cases developing the standard of disclosure, the claims related to a failure to disclose a risk: *Sidaway v Board of Governors of the Bethlem Royal Hospital* [1985] AC 871; *Pearce v United Bristol Healthcare NHS* Trust [1999] ECC 167 (CA); *Montgomery* (n 2). Cases may also involve a failure to disclose alternative treatments which did not carry the risk that materialised, for example, *Montgomery* (n 2).

5 *Chester* (n 2).

6 The ‘but for’ test in causation requires the claimant to prove, on the balance of probabilities, that but for the defendant’s negligence, the harm suffered by the claimant would not have occurred: *Barnett v Chelsea and Kensington Hospital Management Committee* [1969] 1 QB 428.

7 See *Chester* (n 2) [63] citing: *Smith v Barking, Havering and Brentwood Health Authority* [1994] 5 Med LR 285; *Smith v Salford Health Authority* [1994] 5 Med LR 321; *McAllister v Lewisham and North Southwark Health Authority* [1994] 5 Med LR 343; *Smith v Tunbridge Wells Health Authority* [1994] 5 Med LR 334.

8 *Chester* (n 2) [39, 40].

9 ibid [22, 81].

10 See Tamsyn Clark and Donal Nolan, ‘A Critique of *Chester v Afshar*’ [2014] 13 *OJLS* 659. 600, which captures the key articles welcoming and disapproving of the decision in *Chester*, as well as offering its own analysis.

11 *Duce v Worcestershire Acute Hospitals NHS Trust* [2018] EWCA Civ 1307; *Meiklejohn v (1) St George’s Healthcare NHS Trust and (2) Homerton University Hospital NHS Foundation Trust* {2014] EWCA Civ 120.

12 *Correia v University Hospital of North Staffordshire NHS Trust* [2017] EWCA Civ 356.

13 *Diamond v Royal Devon and Exeter NHS Foundation Trust* [2019] EWCA Civ 585.

14 John Coggon, ‘Varied and Principled Understandings of Autonomy in English Law: Justifiable Inconsistency or Blinkered Moralism?’ (2007) 15(3) Health Care Anal 235.

15 Craig Purshouse, ‘How Should Autonomy Be Defined in Medical Negligence Cases?’ (2015) 10(4) Clinical Ethics 107, 111.

16 Her original claim also alleged negligent performance of the operation but this was unsuccessful at trial and that decision was not appealed: *Chester* (n 2) [4].

17 *Chester* (n 2) [1].

18 N7.

19 *Chester* (n 2) [85] per Lord Hope.

20 ibid [24] per Lord Steyn.

21 Jane Stapleton, ‘Occam’s Razor Reveals an Orthodox Basis for *Chester v Afshar* [2006] *LQR* 426, 429 and 446.

22 Sarah Green, ‘Coherence of Medical Negligence Cases: A Game of Doctors and Purses’ (2006) 14(1) Med L Rev 1, 4.

23 ibid 9–10.

24 N11.

25 *Duce* ibid [84].

26 ibid.

27 ibid [85–86].

28 ibid [88]. Clark and Nolan also make this point (n10) 675–76.

29 *Duce* ibid [92].

30 N2 [96–105].

31 *Shaw v Kovac* [2017] EWCA Civ 1028.

32 *Duce* (n 11) [81].

33 *Meiklejohn* (n 11) [34].

34 No case report is available for this decision so the summary of the County Court decision has been drawn from the Court of Appeal judgment (n12).

35 N12 [11–12].

36 N12 [26–27].

37 N3.

38 N12 [26].

39 N12 [24].

40 N12 [28].

41 *Diamond v Royal Devon and Exeter NHS Foundation Trust* [2017] EWHC 1495 (QB) [30–37].

42 ibid [42].

43 ibid [43].

44 ibid [49].

45 ibid [50, 54].

46 ibid [52–53].

47 ibid [55–59].

48 N13 [12].

49 Manning says this reflects an application of the test of materiality from *Montgomery* (n2) which was wrongly applied to causation in *Diamond*: Joanna M. Manning, ‘Oh What an Unholy Mesh! Diamond v Royal Devon and Exeter NHS Foundation Trust’ [2019] EWCA Civ 585 (2019) 27(3) Med Law Rev 519, 523.

50 N13 [21–23].

51 ibid [29–32].

52 ibid [41].

53 Clarke and Nolan (n10) describe these three scenarios as ‘no difference’, no operation’, and ‘delayed operation’ (661).

54 This reflects what Heywood and Miola have previously termed a hybrid subjective-objective test of causation as adopted in *Smith* (n7): Rob Heywood and José Miola, ‘The Changing Face of Preoperative Disclosure: Placing the Patient at the Heart of the Matter’ [2017] 133 LQR 296, 301.

55 Gerald Dworkin, *The Theory and Practice of Autonomy* (CUP 1988) 108.

56 Coggon (n14).

57 ibid 240.

58 ibid.

59 *Chester v Afshar* (No 1997 C No 198. HQ 9902242) Official Transcript [64].

60 Coggon (n14) 241.

61 ibid 253.

62 Purshouse (n15) 110–11.

63 ibid 112.

64 Coggon (n14) 246.

65 *Re A (Children) (Conjoined Twins: Surgical Separation)* [2001] Fam 147: ‘This court is a court of law, not of morals’ 155, per Ward LJ.

